# Treatment of allergic asthma: Modulation of Th2 cells and their responses

**DOI:** 10.1186/1465-9921-12-114

**Published:** 2011-08-25

**Authors:** Berislav Bosnjak, Barbara Stelzmueller, Klaus J Erb, Michelle M Epstein

**Affiliations:** 1 Department of Dermatology, DIAID, Experimental Allergy Laboratory, Medical University of Vienna, Vienna, Austria; 2 BoerhingerIngelheim Pharma, Respiratory Diseases Research, Biberach an der Riss, Germany

## Abstract

Atopic asthma is a chronic inflammatory pulmonary disease characterised by recurrent episodes of wheezy, laboured breathing with an underlying Th2 cell-mediated inflammatory response in the airways. It is currently treated and, more or less, controlled depending on severity, with bronchodilators e.g. long-acting beta agonists and long-acting muscarinic antagonists or anti-inflammatory drugs such as corticosteroids (inhaled or oral), leukotriene modifiers, theophyline and anti-IgE therapy. Unfortunately, none of these treatments are curative and some asthmatic patients do not respond to intense anti-inflammatory therapies. Additionally, the use of long-term oral steroids has many undesired side effects. For this reason, novel and more effective drugs are needed. In this review, we focus on the CD4+ Th2 cells and their products as targets for the development of new drugs to add to the current armamentarium as adjuncts or as potential stand-alone treatments for allergic asthma. We argue that in early disease, the reduction or elimination of allergen-specific Th2 cells will reduce the consequences of repeated allergic inflammatory responses such as lung remodelling without causing generalised immunosuppression.

## Introduction

Asthma is a serious chronic inflammatory lung disease characterised by recurrent episodes of wheezy laboured breathing with prolonged expiration accompanied by dry coughing and viscous mucus. These symptoms result from bronchoconstriction, bronchial mucosal thickening by oedema, eosinophilic infiltration, bronchial wall remodelling and excessive mucus production with plugging of the conducting airways in the lungs. These airway changes lead to increased bronchial hyperreactivity to a variety of allergic and non-allergic stimuli. Obstruction is usually reversible, either spontaneously or in response to appropriate therapy. Asthma affects approximately 300 million people worldwide and can be fatal. Atopic or allergic asthma generally occurs in childhood or young adulthood (under the age of 40) in about 70-80% of cases and is caused by common allergens e.g. pollens, house dust, animal dander, inhalants, foods, drugs and occupationally encountered dust. Atopic asthma is characterised by detectable allergen-specific IgE and a positive skin test upon allergen provocation. The most severe chronic refractory asthma accounts for 5-10% of adults with asthma and is characterised by persistent symptoms and frequent exacerbations, despite treatment with high dose inhaled and/or oral corticosteroids and inhaled β2 adrenoceptor agonists. These patients are at greater risk of fatal and near-fatal exacerbations and display serious unremitting symptoms, resulting in a considerable impact on quality of life, disproportionate use of health care resources and adverse effects from regular systemic steroid use.

**Figure 1:**
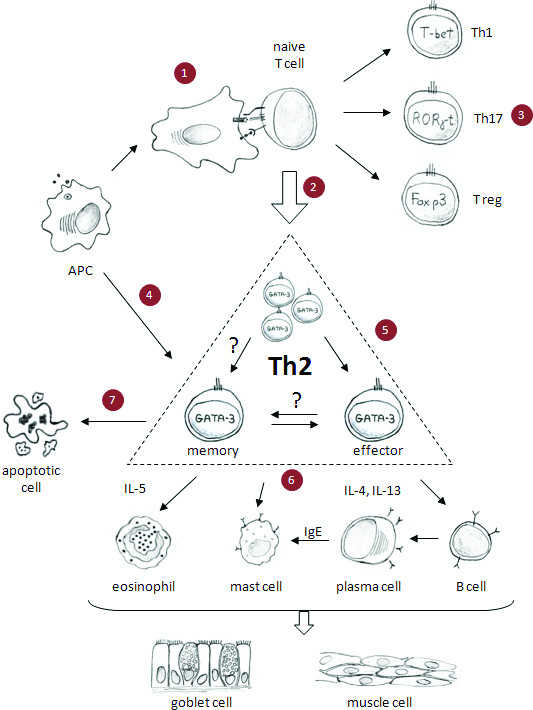
**Helper Th2 cells play a central role in allergic asthma and could be targeted through individual allergic immune processes**. (**1**) Allergen handling and presentation by activated APC to naïve CD4+ T cells induces their activation. (**2**) Activated naïve CD4+ T cells differentiate to Th2 cells, or (**3**) possibly to other types of helper T cells e.g. Th1, Th17 or Treg cells. (**4**) Secondary exposure to allergen leads to Th2 cell activation, (**5**) as well as their migration into the lungs. (**6**) Activated Th2 cell-mediated asthma is caused in part by the secretion of interleukins e.g. IL-4, IL-5 and IL-13. These cytokines stimulate B cell activation and IgE secretion. Th2 cell cytokines and IgE activate cells of the innate immune system e.g. eosinophils, mast cells, etc. causing the release of vasoactive, pro-inflammatory mediators, smooth muscle contraction, mucus hypersecretion, oedema and, eventually, airway remodelling. (**7**) Homeostasis and survival of memory T cells in the lymph nodes and lungs perpetuates disease. Interruption of these molecular and cellular targets may reduce symptoms and pathological consequences of allergic asthma.

The allergic immune response is a complex process beginning with the activation of allergen-specific Th2 cells by antigen presenting cells (APCs) followed by their proliferation, cytokine production, helper functions and the emergence of memory cells (Figure 1). The resulting pathophysiological response includes lung eosinophilic inflammation, oedema, smooth muscle contraction and increased mucus production, resulting in airway obstruction and eventual lung damage. Numerous experimental models and clinical studies support a central role of allergen-specific Th2 cells in pathophysiological responses [1-4]. Although much is known about the pathogenesis of the disease, the mechanisms underlying Th2 cell differentiation and perpetuation remain unclear. Allergen-specific memory Th2 cells take up long-term residence within experimental mice after recovering from a single episode allergic asthma [5] illustrated by the maintenance of elevated serum allergen-specific IgG1 and persistent inflammatory chronic lung infiltrates. Asthma exacerbations are induced by respiratory tract allergen challenge leading to pathology resembling patients [6-8]. A reduction or elimination of specific Th2 responses permits the treatment of disease without causing generalised immunosuppression and makes it a prime target for disease abrogation. Although current asthma therapies (especially inhaled corticosteroids and β2-agonists) efficiently control the disease, development of novel drugs is crucial for disease control in patients with severe, corticosteroid-insensitive asthma, as well as for improvement of existing therapies in terms of a more favourable side effect profile [9]. Additionally, the use of highly active drugs that reduce disease in the early stages may obviate the need for high dose steroids later on and may reduce the potential for unremitting, steroid-resistant disease. Current asthma therapies do not cure the disease and symptoms return soon after treatment is terminated. Treatment in the late stages of chronic, severe, unremitting allergic asthma may be too late. It is therefore, important to start treatment early to reduce disease. In the early stages of disease, allergen-specific Th2 memory cells appear to play an important role in initiating the immune response against the offending allergen. Eliminating these pathogenic cells at an early stage may lead to complete disease remission. There is a myriad of strategies to eliminate Th2 memory cells that are promising. This review focuses on these targets during the evolution of the Th2-mediated allergic immune response from allergen presentation to activation and survival of Th2 memory cells (Figure 1).

### Improvement of existing anti-Th2 cell therapies

Inhaled and oral corticosteroids, leukotriene modifiers, theophyline, anti-IgE and specific allergen immunotherapy (AI) are well-established treatments for asthma [10]. Of these therapies, only AI specifically targets Th2 cells [11]. AI is thought to function by either skewing the allergic Th2 response towards Th1 immunity or generating regulatory T cells (Tregs) [12,13]. While the mechanism remains controversial, AI is effective in a subset of patients. Classical immunotherapy or "allergy shots" in the last years is evolving towards non-injectable forms like subcutaneous and sublingual immunotherapy [13,14]. Progress in AI focuses on the dose and nature of the allergens, with higher allergen doses improving AI effectiveness [15] and chemically modified allergens (allergoids) increasing efficacy [14,16]. The production of recombinant allergens of common allergens from DNA sequences that can be mutated, fragmented or chimerised leads to efficient hypoallergenic mixtures of allergens for treatment [14,17,18]. Additionally important is the ability of producing T cell epitopes without B cell epitopes, which reduces adverse reactions [12,16,17] or new technologies like covalently linked T cell epitopes [14], DNA vaccines encoding allergens [19], production of fusion proteins to increase allergen presentation [20], or expression of recombinant allergens in lactic bacteria able to colonise the gut [21]. Equally promising is the production of random peptide libraries to determine structural equivalents, so called mimotopes [14], producing shorter peptides [16,22], though patients may develop de novo IgE antibodies against the treatment peptide. Although some novel adjuvants such as monophosphoryl lipid A from *Salmonella minnesota *[12,23] or heat killed or live *Mycobacterium tuberculosis *did not meet expectations in clinical trials [14], other adjuvants like fusion proteins with bacterial surface layer components [14] and  cytosine-guanine dinucleotides (CpG) oligonucleotides (CpG-ODNs) [9,12,23], as well as routes of allergen delivery, in oral microencapsulated forms [24] or embedded in nanoparticles [23], are being explored.

### Strategies to modulate antigen presentation and Th2 cell activation

Dendritic cells (DCs) expressing CD11c^+^CD11b^+ ^[25], CD16^+ ^[26], CD141^+ ^[27] or CD8α [28] predispose to allergic asthma. Sputum and bronchial biopsies of asthmatic patients contain higher DC numbers in comparison to healthy individuals [29,30] and are increased after allergen exposure [31]. Asthmatic DCs differ in cytokine, prostaglandin (PG), and chemokine synthesis and costimulatory molecule expression compared to healthy controls [32-34]. In addition, allergen-pulsed DCs from asthmatic patients, but not healthy controls, preferentially stimulate T- cells to produce IL-4 [35]. DCs from asthmatics produce high amounts of PGE2 [34], which decreases IL-12 [36] and increases CCL17 and CCL22 production [37] from DCs causing the polarisation of DCs, which promote Th2 cell differentiation and recruitment. Recently, thymic stromal lymphopoietin (TSLP) has emerged as a key mediator, which promotes DC-induced Th2 differentiation through the interaction of OX40:OX40L [38,39]. Inhibition of DC-mediated antigen presentation represents a suitable treatment option for allergic diseases. While DCs are the most potent APCs, other cells also contribute to antigen presentation and may provide useful targets. Table 1 illustrates the cell type, target and mechanism of action for compounds and biologicals that reduce antigen presentation to Th2 cells and their subsequent activation.

**Table 1:** Antigen presenting cell targets

Cell type	Target	Intervention example	Mechanism of action and effects	Comments
Dendritic cell	Peroxisome proliferator-activated receptor gamma	Rosiglitazone and ciglitazone	Decrease CCR7 expression on DCs and diminishes migration [144,145]	-
	Sphingosine 1-phosphate inhibitor	FTY720	Sequesters lymphocytes in secondary lymphoid organs; inhibits T cell migration to the draining lymph nodes [146-;] suppresses eosinophilic airway inflammation and AHR, reduced Th2 cell generation [147,148], generalised immunosuppression [149]	In clinical study for moderate asthma (ClinicalTrials.gov identifier: NCT00785083)
	Thymic stromal lymphopoietin (TSLP)	Anti-TSLP antibodies [39]	TSLP skews DCs to express high levels of OX40 ligand, which promotes the generation of Th2 cells [38]; its inhibition prevents Th2-mediated airway inflammation in mice [39]	-
	CCL2	CCR2 antagonists [150]	Overexpressed in lung and increased DC recruitment in allergic asthma [151,152]	CCR2 is involved in migration of other immune cells as well
	CD80/86 costimulation	D prostanoid 1 receptor agonist [153], aerosolised CD86 antisense oligonucleotide [154] or suplatast tosilate [155],	Reduce allergic disease in mice models of acute asthma	CD80/86 co-stimulation does not contribute to recall responses of effector Th2 cells [156] and might not be useful for the treatment of established disease
	OX40L	Anti-OX40L Ab	Blocks Th2 cell infiltration, cytokine secretion, IgE production and Th2 inflammation in mouse and non-human primate models [157]	-
	Programmed death-1 (PD-1) and PD1 ligands	None so far	PD-1 and its ligands regulate T cell activation and differentiation and affect asthmatic responses [158]	-
Macrophage	Anti-A1 adenosine receptors	A1 adenosine receptor modulators	Anti-inflammatory [159]	Gene expression and function depends on polarisation (classical vs. alternative activation) [160]
	Unknown	Water-soluble chitosan	Suppresses allergic asthma in mice [161]	
	Unknown	Mycolic acid	Modulates airway macrophage function to suppress allergic inflammation in mice [162]	
Basophil	Specific target unknown so far	N/A	CD49b^+^FcεR^+ ^basophils migrate from blood to lymph nodes, where they present processed antigen to T cells in the context of MHC class II molecules and induce Th2 type polarisation through secretion of IL-4 [163-166]	Recently, the role of basophils in Th2 immunity was disputed in favour of inflammatory DCs [167,168]

### Interference with Th2 differentiation and activation

Antigen presentation induces clonal expansion and differentiation of naïve Th cells into mature Th1, Th2, Th17 or inducible Tregs [reviewed in [40]]. Th2 cell polarisation is mediated by transcription factors, including GATA-3, which are crucial for Th2 lineage commitment. Initial signals that drive Th2 differentiation induce expression of the GATA-3 [41], which mediates Th2 differentiation by inducing chromatin remodelling of Th2 gene loci, direct transactivation of Th2 gene expression and inhibition of IFNγ expression [42]. Furthermore, GATA-3 expression must be sustained to maintain a Th2 phenotype [42,43]. Beside other important factors, microRNAs have recently emerged as regulators of gene expression during differentiation and function [reviewed in [44,45]]. Numerous microRNAs play important roles in asthma [46] and selective inhibition of these molecules can be utilised to specifically target development of Th2 cells. Examples of other signal transduction pathway targets and their inhibitors are listed in Tables 2 and 3. Unfortunately, most of these targets are not selectively expressed in Th2 cells and their inhibitors have broad immunosuppressive effects.

**Table 2:** Strategies to inhibit Th2 cell differentiation

Target	Mechanism	Intervention example	Effect	Comment
GATA-3	Development of Th2 cells [169]	Local treatment with GATA-3 antisense oligonucleotides [170] or RNA interference delivered by a lentiviral vector [171]	Inhibits allergen-induced asthma	Important for T cell development, its inhibition could cause immunosuppression [169]
STAT3	Important for differentiation of Th2 cells [172]	Selective small molecule inhibitors [173]	Inhibits allergen-induced asthma	-
STAT5a	Important for differentiation of Th2 cells	None known	STAT-5a deficient mice have decreased IL-5 production and Th2 and eosinophil recruitment in mouse model of asthma [174]	Also important for development of inducible Tregs [175]
STAT6	Important for differentiation of Th2 cells	Selective small molecule inhibitors [176] or RNA interference [177] of STAT6	Suppresses Th2 responses *in vitro *and in animal models	-
Notch	Binds to the promoter of GATA-3 and regulates its transcription [178,179]	Gamma-secretase inhibitor (GSI) [180]	Selective inhibition of Th2, but not Th1 responses [181]	Involved in development of many other leukocytes and organs [182,183]
c-Maf	Transcription factor expressed at high levels in Th2 cells [184,185]	So-Cheong-Ryong-Tang (a Korean traditional medicine; [186]) or KR62890 (agonist of peroxisome proliferator-activated receptor γ; [187])	Inhibits Th2 cell functions	Inhibits Th-17 and Treg function
Gfi-1, Dec2, ROG and Bcl-6	Transcription repressors important for Th2 cell development [188-192]	None known	N/A	-
SOCS-3	Inhibitor of cytokine signalling pathways [193]	None known	SOCS-3 blocks Th1 cell development and is preferentially expressed in Th2 cells [194]	Appears to be involved in Treg and/or Th17 cell development [195]
SOCS-5	Inhibitor of cytokine signalling pathways [193]	None known	Preferentially expressed in Th1 cells and prevents Th2 cell development [196]	Its over-expression in T cells enhances airway inflammation and AHR [197]
miRNA-16, miRNA-21, miRNA-126	Up-regulated in lung tissue after allergen challenge in mouse models of asthma [198,199]	Anti-miRNA-126 antagomir (small synthetic RNA molecule with modified backbone for degradation prevention) [199]	Prevents allergen-induced airway hyperreactivity and reduces allergic inflammation	-

**Table 3:** Interference with Th2 signal transduction pathway and their inhibitors

Class	Examples of inhibitor(s)	Effect	Reference*
EGF receptor inhibitor	Gefitinib	Reduces the cell counts and Th2 cytokine levels in an OVA-challenged mouse model of allergic asthma	[200]
Syk inhibitors	BAY 61-3606	Inhibits disease signs in a mouse model of asthma	[201]
	R112	Reduces allergic rhinitis upon intranasal administration	[202]
JAK3 inhibitors	CP690550	Blocks expression and signalling of IL-2, IL-4 and IL-13	[203]
	WHI-P131 and WHI-P97	Interferes with inflammatory mediators and mast cell degranulation in animal models of asthma	[204,205]
p38 MAPK/ERK inhibitor	U0126	Inhibits airway and lung inflammation in mouses model of asthma Role of p38 in steroid resistant asthma patients is investigated in a clinical trial	[206] NCT00676572
Inhaled p38 MAPK antisense oligonucleotide	ISIS101757	Inhibits allergic immunity in mice	[207]
p38α inhibitors	BIRB796, SB203580 and RWJ67657	Inhibits airway and lung inflammation in mouse models of asthma	[208-210]
JNK inhibitor	SP600125	Inhibits T cell cytokine production and lung inflammation in mouse models of asthma	[211,212]
Inhibitor of adenosine A1, A2b and A3 receptors, p38 MAPK and PDE4D	CGH2466	Inhibits allergic asthma in mice	[213]
PI3K inhibitors	Wortmannin and Ly294002	Inhibits allergic asthma in mice	[214,215]
Inhibitor of IkappaB kinase-2 (IKK-2)	N/A	Reduces allergen-induced airway inflammation and AHR in animal models of asthma	[216,217]
IkappaB ubiquitination inhibitor	GS143	Represses Th2, but not Th1 differentiation after allergen challenge in a mouse model of allergic asthma	[218]
Selective PDE4 inhibitors	GSK256066, MK-0359	Inhibits the fall in lung function in patients with asthma caused by inhaled allergen challenge	[219,220]
PDE3 and PDE4 inhibitors	RPL554	Inhibits eosinophil recruitment following antigen challenge in guinea pigs	[221]

* Numbers starting with NTC represent clinical study code from http://clinicaltrials.gov/

### Modulation of effector cytokines

The interplay between cells and cytokines involved in Th2-mediated disease is complex. Th2 cells secrete and express a variety of cytokines and receptors [40]. In the past decade, mAbs targeting the most prominent Th2 cytokines, IL-4, IL-5 and IL-13 have had variable success in clinical trials and the perception is that effectiveness will be improved by inhibiting two or all of them simultaneously. Furthermore, additional cytokines including IL-9 and IL-31 are secreted by Th2 cells and might represent novel or additive targets. Moreover, cytokines secreted by other cells such as Th1, Th17 and Tregs may suppress Th2 cell function. Importantly, augmenting suppressive effects and inhibiting disease-promoting effects of T cells may lead to new compounds. Table 4 illustrates examples of cytokines secreted by Th2 cells, have direct effects on Th2 differentiation or are involved in differentiation of other helper T cell subtypes that could inhibit Th2 cells.

**Table 4:** Effector cytokines as targets

Cytokine	Relation to Th2 cells in asthma	References	Was the target used in clinical trials in asthma?	Clinical study, Reference*
IL-2	Important for survival of mature Tregs Required for generation of effector and survival of memory T cells	[175] [222]	Yes, daclizumab targeting its soluble IL-2 receptor CD25, improves FEV_1 _and reduced daily asthma symptoms	NCT00028288
IL-3	Secreted by Th2 cells, regulates eosinophil and basophil differentiation, migration and survival Inhibition of IL-3/IL-5/GM-CSF common β receptor inhibits Th2 differentiation	[223,224] [225]	No	-
IL-4	Crucial for Th2 cell differentiation Induction of IgE production of B cells	[226]	Yes, numerous mAbs and other compounds, development of most mAbs was discontinued, pitrakinra (IL-4 mutant protein binding to IL-4 and IL-13 receptors) improves lung function, stabilises asthma symptom scores and reduces beta-agonist use	[9,11,227-229], NCT00801853, NCT00941577
IL-5	Th2 cell cytokine involved in eosinophil differentiation, maturation, recruitment and survival	[230,231]	Yes, does not inhibit eosinophilia or AHR, but new indications suggest use in difficult-to-treat and severe asthma	[232-234], NCT01000506, NCT00292877
IL-6	Polarises CD4+ T cells to Th2 or Th17 subtype Soluble IL-6 receptor induces apoptosis of Th2 cells in the lungs & induces Tregs	[235,236] [237]	No	-
IL-9	Secreted by Th2 cells Over expression in mice enhances inflammation and AHR	[238] [239,240]	Yes, appears to have acceptable safety profile and to decrease FEV_1_	[241,242]
IL-10	Secreted by Th2 cells and some Tregs, plays multiple roles in the immune processes	[243]	No	-
IL-12	Essential for differentiation, proliferation and activation of Th1 cells Suppresses Th2 immune responses in murine models	[244] [245]	Yes, reduction in the number of circulating blood eosinophils, but not sputum eosinophilia, the late-phase response or airway hyper-responsiveness	[246]
IL-13	Involved in lung inflammation, mucus hypersectretion, subepithelial fibrosis and eotaxin production	[247]	Yes, clinical trials for numerous mAbs are in progress; pitrakinra (IL-4 mutant protein binding to IL-4 and IL-13 receptors) improves lung function, stabilises asthma symptom scores and reduces beta-agonist use	[229,248,249], (NCT00873860, NCT00801853, NCT00941577)
IL-15	Th1 cytokine that appears to counterbalance Th2 immune response	[250]	No	-
IL-17A	Implicated in infiltration of neutrophils after allergen exposure Might regulate established Th2 response	[251] [252]	No	-
IL-17F	Implicated in infiltration of neutrophils after allergen exposure	[251]	No	-
IL-18	Cytokine involved in Th1 and Th2 immunity Delivery of IL-18 gene reduced allergic inflammation in a mouse asthma model	[253] [254]	No	-
IL-19	Produced by epithelial cells and mediates IL-4, IL-5, IL-10 and IL-13 production	[255,256]	No	-
IL-21	Secreted by CD4+ T cells Involved in proliferation, differentiation and regulation of T cells, B cells, DCs and natural killer cells Stimulates IgG responses instead of IgE	[23,257]	No	-
IL-22	Required for the onset of allergic asthma in mice, but negatively regulates acute inflammation in lungs	[258]	No	-
IL-23	Lung-specific expression enhances allergen-induced inflammation, mucus hyperproduction and AHR Its inhibition protects against allergic asthma in mice	[259] [260]	No	-
IL-25	Induces Th2 immunity, enhances Th2 cell survival and stimulates Th2 cytokine secretion Its inhibition prevents inflammation in mouse models of asthma	[261,262]	No	-
IL-27	Th1 cytokine decreases Th2 response in murine models of asthma	[263]	No	-
IL-31	Secreted by Th2 cells, expressed at higher levels in asthmatic patients	[264,265]	No	-
IL-33	IL-33 receptor, ST2, is a marker for Th2 cells IL-33 activates Th2 cells	[266,267] [268]	No	-
IFN-γ	Th1 cytokine that inhibits Th2 cell polarisation *in vitro* Appears to be involved in pathogenesis of severe allergic asthma	[40] [269,270]	Yes, but treatment did not improve monitored clinical parameters	[271]
TGF-ß	TGF-ß inhibits expression of transcription factor GATA-3 Its neutralisation exacerbates or has no effect on inflammatory responses in mouse models of asthma	[272] [273,274]	No	-
TNF-α	Pleiotropic cytokine, chemoattractant for eosinophils and contributes to the activation of T cells	[275]	Yes, divergent results, severe side-effects	[276,277]

* Numbers starting with NTC represent clinical study code from http://clinicaltrials.gov/

### Interference of Th2 cell homing and adhesion

Chemokine-chemokine receptors (CKRs) are a complex system of 42 molecules and 19 receptors that orchestrate leukocyte migration in physiologic and pathologic conditions [47]. Among CKRs, CCR4, CCR8, CXCR4 and CCR3 appear to be selectively expressed on Th2 lymphocytes [48,49] making them potentially important specific Th2 cell targets. CCR4 regulates chemotaxis of Th2 cells and its ligands CCL17 and CCL22 are elevated in allergic asthma [50,51]. Hence, selective CCR4 antagonists, such as bipiperidinyl carboxylic acid amides, or antibodies directed against CCR4 ligands could be promising treatments [9,50,51]. However, CCR4 is also expressed on Tregs and cells with either Th1 or Th2 potential [52] leading to CCR4 inhibitors causing immunosuppressive effects. CCR8 expression also appears to be increased in lung and airway Th2 cells in asthmatic patients [53]. Airway eosinophilia and  airway hyperresponsiveness (AHR), however, are not diminished in CCR8^-/- ^mice [54] and adoptively transferred Th2 cells not expressing CCR8 accumulate in the lungs [55]. Despite these contrasting results, several CCR8 agonists [56] and antagonists [57] are in development and might help to clarify the role of CCR8 in disease pathogenesis. CXCR4 is also involved in Th2 cell migration into the lungs [58] and treatment of allergic mice with selective CXCR4 inhibitors significantly reduces AHR and inflammatory responses [59,60], supporting the further development of CXCR4 antagonists for asthma treatment. CCR3, which regulates eosinophil and mast cell accumulation into the lungs [61], is expressed on Th2 lymphocytes [62]. CCR3 inhibition is a promising Th2 cell target that reduces innate and adaptive allergic inflammation [63]. TPI ASM8 is a compound that contains modified antisense oligonucleotides targeting CCR3 and the common beta chain of the receptors of GM-CSF, IL-5 and IL-13, decreases airway inflammation in humans after allergen exposure and is under clinical evaluation [64]. Other CKRs that appear to regulate CD4+ T cell homing to the lungs in asthma include CCR5, CCR6, CCR7 and CXCR3 [65-67]. CCR7 is a CKR expressed on a large number of naïve and memory T cells [47] and therefore does not represent suitable target. Expression of CCR5, CCR6 and CXCR3 is related to Th1 (CXCR3 and CCR5) [48,49] or Th17 (CCR6) cells [68]. Thus, it is possible that CKR agonists, rather than antagonists, might inhibit Th2 cells in asthma. Importantly, the chemokine system is highly redundant with promiscuous chemokine-CKR interactions, suggesting that a single chemokine or CKR could have compensatory mechanisms leading to unexpected side effects. Moreover, blocking of a single chemokine or CKR might also not have an effect due to this redundancy.

CRTH2 is a mediator involved in the migration and activation of basophils, eosinophils and Th2 cells [69,70]. CRTH2 inhibition leads to attenuated airway hyperreactivity and inflammation in animal models [71]. Ramatroban, a dual thrombroxane A2 receptor (TP) and CRTH2 receptor antagonist, suppresses eosinophil chemotaxis *in vitro *and *in vivo *and is approved for the treatment of allergic rhinitis in Japan [72]. Numerous other CRTH2 antagonists, such as 4-aminotetrahyrochinoline derivatives or indoleacetic acid derivatives, are currently under development [69,70,72] and OC000459 is in clinical trials for the treatment of allergic asthma (ClinicalTrials.gov identifier: NCT01057927, NCT00890877). The CRTH2 receptor is a DP2 receptor. Biological effects of PGD2 and PGH2 are mediated by D prostanoid receptor 1 (DP1) and CRTH2 (DP2). PGD2 activates DP1, thereby affecting NK cells and their cytokine production into a profile more favourable for Th2 skewing [73]. PGH2 is implicated in the accumulation of CRTH2+ cells at sites of inflammation [74]. Additionally, as discussed above, PGE2 polarises DCs to promote Th2 cell differentiation and recruitment [34,36,37]. These effects of PGE2 seem to be mediated by PGE2 receptor type 2 (EP2) and type 4 (EP4) [75]. Therefore, PGs and CRTH2 appear to be promising Th2 cell-specific targets.

While homing receptors are important for Th2 cell migration, several adhesion molecules also play a role. For example, intercellular adhesion molecule (ICAM)-1 and ICAM-2 play important roles in T cell migration in the lungs [76] and ICAM-1 deficiency reduces leukocyte infiltration into the airways, as well as IL-4 and IL-5 concentration in bronchoalveolar lavage fluid [77]. Additionally, VCAM-1 plays a role in eosinophil migration and activation in addition to T cell trafficking [78]. There are no clinical data to date for mAbs against ICAM-1 or VCAM-1 in the treatment of asthma. Other potential adhesion targets include VLA-4 (α4ß1 integrin) [79] or P-, E- and L-selectins [80]. Natalizumab blocks both α4ß1 and α4ß7 integrins, but was discontinued due to severe side -effects [81]. Novel α4 integrin mAb LLP2A reduces AHR and inflammation in mouse allergic asthma [82]. Unfortunately, initial results with VLA-4 antagonist GW559090 were disappointing [83], but newer and safer alternative VLA-4 antagonists are in development [84-86]. Lastly, a pan-selectin inhibitor is currently in phase IIa clinical trials for COPD, might also be promising for asthma [81]. None of these adhesion molecules is selectively expressed on Th2 cells.

The anticoagulant heparin has anti-inflammatory properties that inhibit leukocyte extravasation [87]. IVX-0142 is a heparin-derived hypersulfated disaccharide that appears to be well-tolerated and shows a trend towards attenuation of asthmatic responses, but does not affect AHR [88]. Additional studies are needed to evaluate effects of these molecules on Th2 cells.

### Inhibition of long-lived Th2 memory cells

It is possible that long-lived Th2 memory cells establish anti-apoptotic mechanisms for long-term maintenance, which when inhibited may result in cell death. Interfering with mechanisms for their longevity in the lungs may eliminate Th2 cells. Corticosteroids [89], calcineurin inhibitors [90] and the cysteine leukotriene receptor antagonist montelukast [89] have pro-apoptotic effects on activated T cells, one of the many mechanisms that lead to their effectiveness in asthma. CX3CR1 seems to provide a survival signal for lung Th2 and Th1 cells, which when inhibited reduces allergic inflammation [67]. T cells from p53-deficient mice have decreased apoptosis and increased Th2 differentiation [91], cytoxic lymphocyte antigen-4 (CTLA-4) promotes T cell apoptosis [92,93] and CTLA-4-deficient Th cells are directed towards Th2 differentiation [94]. Additionally, the ratio of anti-apoptotic protein Bcl-2 over pro-apoptotic protein Bax in peripheral blood lymphocytes of asthmatic patients is increased in comparison to healthy controls [95]. Interestingly, Th2 cells express less Fas ligand (FasL) and are more resistant to apoptosis than other Th subtypes [96,97]. Moreover, the Th2 cytokine IL-4 reduces FasL, while Th1 cytokines IFNγ, TGFβ and IL-2 increase FasL expression [89]. Regulation of FasL plays an important role because FasL-expressing T cells are pivotal during the resolution of airway inflammation [98] and intratracheal delivery of DCs co-transfected with FasL and allergen genes before allergen challenge-induced T cell apoptosis and decreased airway inflammation in mice [99]. Induction of Fas expression on Th2 cells might be a possible treatment approach that would decrease their survival in the lungs despite the fact that Th2 cells are somewhat resistant to Fas-induced apoptosis. An additional important pathway for apoptosis in T cells involves granzyme B, which is critical for activation-induced cell death [100]. Inhibition of granzyme B rescues Th2 cells from apoptosis [100], suggesting that selective activation of granzyme B in Th2 cells might be a novel target. Another possibility is that increased apoptosis of Tregs and their protection from apoptosis might be a method of treating disease but there is little information related to cell death of Tregs in allergic diseases and it is possible that dysregulated apoptosis of Tregs may contribute to allergic asthma [90].

### New categories of targets: Statins and Rho kinases; TIM proteins; Galectins; Siglecs; Arginases; Histone deacetylase inhibitors; Pathogens and Toll-like receptors

Statins are a class of cholesterol lowering drugs that also possess anti-inflammatory and immune properties [101,102]. Simvastatin, Lovastatin and Pravastatin reduced eosinophilia and Th2 cytokines in animal models of asthma [103-105]. Clinical trials evaluating Simvastatin (NCT00792337), Lovastatin (NCT00689806) and Atorvastatin (NCT00463827), are ongoing or completed, but data are not yet available. Some statins exert their action through regulation of Rho kinases [106], which are expressed at high levels in airway smooth muscle and regulate their contractility [107], but inhibition appears to impair lymphocyte cytokine secretion [108].

The genes for the T cell immunoglobulin domain and mucin domain (TIM) proteins are encoded in the T cell and airway phenotype regulator region on chromosome 11 [109]. Initial results indicate that although TIM-1 is involved in Th2 cell differentiation and is associated with Th2-mediated diseases [110], it also regulates Th17 and Treg development. Furthermore, TIM proteins are expressed by other immune- cell types [111]. Because TIM proteins do not exclusively regulate Th2 cells, they are less useful as targets than originally anticipated.

Galectins are β-galactoside-binding proteins that bind to glycan residues on the surface of mammalian cells [112]. Examples are Galectin-3 and -9, which appear to have numerous functions in T cell activation, differentiation and apoptosis [112]. Airway inflammation and challenge is decreased in Galectin-3 knockout mice [113] and intranasal administration of a plasmid encoding Galectin-3 abates chronic airway inflammation in a murine model of asthma [114]. Galectin-9 binds to TIM-3, which is expressed on Th1 cells and is important for protective immunity against microbes [111] and intravenous administration of Galectin-9 suppresses AHR and airway inflammation in a mouse model of asthma [115].

Siglecs are sialic acid-recognising Ig-superfamily lectins [116]. CD33-related Siglecs, which in humans include Siglec-3 and Siglecs-5 through -11, are predominantly found on human leukocytes and involved in innate immunity [116,117]. Mouse Siglec-F, the equivalent of human Siglec-8, is expressed on eosinophils and regulates their apoptosis [118]. Blocking Siglec-F function with a mAb reduces airway and lung eosinophilia in mice [119]. Although Siglec-8 is a promising target directed against eosinophils, human T lymphocytes express little or no siglec molecules [120] and do not appear to be a candidate for inhibiting Th2 cells.

Arginase I and II are cationic amino acid transporters involved in the metabolism of basic amino acids expressed in inflammatory lesions of patients with allergic asthma [121-123]. Arginase gene expression and enzyme activity are enhanced by IL-4 and IL-13 [122,123]. Inhibition of arginase I by RNA interference suppresses IL-13-mediated AHR in a murine model of asthma [124] and inhalation of an arginase inhibitor decreases AHR and airway inflammation in a guinea pig model of asthma [122]. Conversely, deletion of arginase in macrophages impairs their ability to suppress Th2-dependent inflammation and fibrosis [125]. Further research is needed in order to clarify the role of arginases in Th2 immunity.

Histone deacetylases (HDAC) appear to play an important role in cytokine transcription [126]. Corticosteroid signalling requires HDAC2 to suppress inflammatory gene products and HDAC2 activity is diminished in corticosteroid-resistance [9]. HDAC inhibitor Trichostatin A reduces allergic airway inflammation by decreasing expression of the Th2 cytokines, IL-4, IL-5 and IgE [127]. In contrast, HDAC1 appears to be a negative regulator of Th2 cytokine expression [128]. Chromatin modification enzymes might be potential targets for inhibition of Th2-mediated diseases.

Many microbials or their proteins inhibit Th2 immune responses in murine models of asthma by polarising towards Th1 immunity [129-131] or by generating suppressive Tregs [132,133]. Interestingly, microbial agents have both time- and dose-dependent effects on allergic asthma [134,135]. Certain allergens such as dust mite Der p2 and Der f2, bind LPS and are related to the MD-2 protein of the LPS-binding component of the TLR4 signalling complex [136,137], which might influence the induction of Th2 responses demonstrating a potential for microbials augmenting rather than reducing Th2 responses. Alternatively, bacterial DNA or chemically synthesised *de novo *unmethylated CpG are immunostimulatory ligands that bind to TLR9 and induce strong Th1 immune responses [reviewed in [138]]. Such bacterial and synthetic DNA immunostimulatory oligonucleotides (ISS-ODNs) containing CpG motifs suppress Th2 responses during the sensitisation phase or immediately before challenge in experimental asthma [reviewed in [139]]. They have therapeutic and prophylactic properties [140], including suppression of DC migration and co-stimulatory molecule expression and inhibition of IgE-dependent Th2 cytokine release from mast cells and basophils [141,142]. Moreover, ISS-ODNs added to allergen immunotherapy significantly reduce clinical symptoms in patients with asthma [143].

## Conclusions

**Figure 2:**
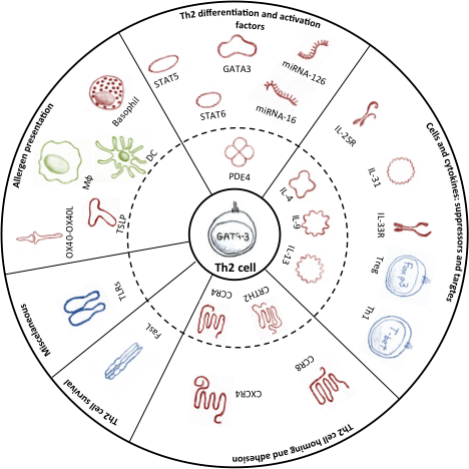
**Promising cellular and molecular target candidates for inhibiting memory Th2 cells in allergic asthma are grouped according to the subchapters of the text**. Targets in the inner circle (dash line) were tested in clinical trials, whereas other targets are still in pre-clinical stages. Treatment targets are divided into the following categories: Red colour indicates target inhibition; Blue colour indicates target activation; and Green colour indicates targets that can be inhibited or activated to inhibit Th2 cells. For details, please refer to the text. Abbreviations: DC - dendritic cell; FasL - Fas ligand; IL - interleukin; IL-25R - interleukin 25 receptor; IL-33R - interleukin 33 receptor; miRNA - microRNA; Mφ - macrophage; PDE - phosphodiesterase; STAT - Signal transducer and activator of transcription; Th1 - helper T cell type 1; Th2 - helper T cell type 2; TLR - Toll like receptor; Treg - regulatory T cell; TSLP - thymic stromal lymphopoietin.

Th2 cells and/or their secreted effector molecules mediate the immune response to allergens and are triggered by exposure to specific allergens leading to allergic asthma. Thus, inhibiting or eliminating Th2 cells is a beneficial strategy for treating asthma as long as generalised immunosuppression is avoided. Additionally, it is especially important to consider targeting Th2 cells early in disease because when disease is chronic additional factors may cause perpetuation. Although there are a myriad of potential Th2 targets (Figure 2), the optimal, most effective anti-Th2 cell target for the clinic remains elusive.

Aside from anti-IgE therapy for severe asthma, there are no major new drugs for the treatment of asthma in the last 20 years. The latest research in allergic asthma that has elucidated key factors governing Th2 immunity and identified potential targets is predominantly from animal models. Now, the challenge is to discover candidates, which best translate from animal models to patients. However, choosing the most effective new drug target candidate is especially difficult because human data is often lacking or incomplete. Additionally, the use of accurate, predictive biomarkers to evaluate Th2-modulating drugs such as FEV1, Quality of Life, reduction in steroid use, decrease allergen-induced late phase response and others are important to ensure that the efficacy/adverse effect profiles are standardised and enable easier decision-making for the best candidates. We would argue that the most promising new compounds for the clinic are those in which proof of concept in patients is established e.g. anti-IL-13 antibodies and CRTH2 antagonists. Other candidates currently tested in the clinic are anti-IL-5, anti-IL-4 and anti-IL-9 compounds and CCR4 antagonists. However, based on available human data, we suggest that the epithelial cell-derived cytokines TSLP, IL-25 and IL-33, which drive Th2 responses are the most promising candidates, with TLSP the clear frontrunner.

Steroids are efficient for treating asthma because they inhibit numerous pro-inflammatory responses and induce numerous anti-inflammatory pathways. Thus, targeting a single mediator may not suffice for the treatment of allergic asthma because of the redundant immune and inflammatory pathways involved upon allergen challenge. Thus, we suggest that targeting more than one molecule simultaneously using dual specific antibody/protein platforms to engineer new drugs will be the next major approach in drug discovery. However, while this approach creates a scenario in which numerous targets can be combined, the caveat is that optimal candidates must be carefully chosen. Another important consideration for the therapeutic strategy for allergic asthma is that drugs may need to be developed for specific subtypes of disease in which particular cellular and molecular pathways drive the disease. One example is the anti-IL-5 mAb, which is only effective in asthmatics with very high sputum and lung eosinophil numbers. This example suggests that it is beneficial to better categorise patients and consider personalised medicine based on a clear classification of disease.

These are exciting times for Th2 cell immunology as the results of basic research are defining key molecular and cellular components in the response to allergens. This information is already being converted to targets that are being tested in the clinic. Currently, irrespective of approach, we consider that a successful strategy for the treatment of allergic asthma will include a selective inhibition of Th2 cells with the ultimate aim of eliminating allergen-specific Th2 immune responses. We anticipate that new candidates will be approved in the near future and offer treatment options for patients suffering with asthma and other allergic diseases.

## List of abbreviations

AHR: Airway hyperresponsiveness; AI: Allergen immunotherapy; APC: Antigen presenting cell Bcl: B cell lymphoma; Th: CD4+ T helper; CKR: Chemokine receptor; CRTH2: Chemoattractant receptor-homologous molecule expressed on TH2 cells; CpG: Cytosine-guanine dinucleotides; CpG-ODN: Cytosine-phosphate-guanine oligonucleotides; CTLA-4: Cytoxic lymphocyte antigen- 4; DC: Dendritic cell; DP1: D prostanoid receptor 1; EGF: Epidermal growth factor; ERK: Extracellular signal regulated kinase FasL: Fas ligand; GM-CSF: Granulocyte-macrophage colony-stimulating factor; HDAC: Histone deacetylases; Ig: Immunoglobulin; ISS-ODNs: Immunostimulatory oligodeoxynucleotides; ICAM: Intercellular adhesion molecule; IFN: Interferon; IL: Interleukin; JAK: Janus kinase; JNK: Jun kinase mAb: monoclonal antibody; MAPK: Mitogen-activated protein kinases; PDE: Phosphodiesterase; PI3K: Phosphoinositide 3-kinase; PD-1: Programmed death-1; PG: Prostaglandin; Siglec: Sialic acid binding Ig-like lectins; STAT: Signal transducer and activator of transcription; SOCS: Suppressor of cytokine signalling; Treg: regulatory T cell; TSLP: Thymic stromal lymphopoietin; TLR: Toll-like receptor; TGF: Transforming growth factor; TNF: Tumour necrosis factor; VCAM: Vascular cell adhesion molecule; VLA: Very late antigen.

## Competing interests

**Berislav Bosnjak **- was employee of GlaxoSmithKline Research Centre Zagreb Ltd. until December 2008. No other competing interests.

**Barbara Stelzmüller **- none

**Klaus J. Erb **- is an employee of BoerhingerIngelheim Pharma, Respiratory Diseases Research, Biberach an der Riss, Germany

**Michelle M. Epstein **- received funding from BoerhingerIngelheim Pharma, Respiratory Diseases Research, Biberach an der Riss, Germany for collaborative project

## Authors' contributions

BB - was involved in drafting the manuscript, revising it critically for important intellectual content; and has given final approval of the version to be published.

BS - made substantial contributions to conception of the review, was involved in drafting the manuscript, revising it critically for important intellectual content; and has given final approval of the version to be published.

KE - made substantial contributions to conception of the review, was involved in drafting the manuscript, revising it critically for important intellectual content; and has given final approval of the version to be published.

ME - made substantial contributions to conception of the review, was involved in drafting the manuscript, revising it critically for important intellectual content; and has given final approval of the version to be published.
